# A qualitative analysis of college students’ interest in mHealth solutions

**DOI:** 10.3389/fpubh.2025.1605222

**Published:** 2025-05-30

**Authors:** Leslie Hoglund, Craig M. Becker, Cara Tonn

**Affiliations:** ^1^Department of Health Behavior, Policy, and Management, Joint School of Public Health, Old Dominion University, Norfolk, VA, United States; ^2^Department of Health Education and Promotion, College of Health & Human Performance, East Carolina University, Greenville, NC, United States

**Keywords:** mHealth (mobile health), artificial intelligence, college students, focus group, digital health (DH)

## Abstract

This study explores college students’ perceptions of an AI-driven mHealth application designed to promote well-being. With rising mental health challenges in academic settings, students increasingly seek digital tools that provide holistic support for physical, mental, and financial health. Through focus groups, this qualitative study examines students’ preferences for personalized health tracking, educational content, and flexible reminders within a private, supportive community. Key findings emphasize students’ desire for a balanced, all-in-one app that integrates health and wellness tools without overwhelming them with notifications. Students also highlighted the importance of social media integration for outreach, though concerns were raised about potential stress from competitive online environments. The findings underscore the value of user-centered design in developing mHealth solutions that foster engagement, simplify wellness management, and respect user privacy. This research contributes to understanding how digital platforms can be tailored to support college students’ well-being effectively.

## Introduction

The increasing use of mobile technology to promote wellness among college students is well-documented (1–3). Mobile health (mHealth) applications, especially those powered by artificial intelligence (AI), have shown promise in offering tailored support to encourage positive health behaviors. AI-driven systems like Alexa, Siri or DJ on Spotify provide personalized recommendations based on user behavior, and similar approaches are now emerging in wellness technologies. However, while the potential of mHealth apps is evident, various factors such as perceived usefulness, ease of use, privacy concerns, and personalization can significantly influence user adoption and engagement.

For example, the Unified Theory of Acceptance and Use of Technology (UTAUT) provides a framework that utilizes perceived usefulness, ease of use, and personalization. The study completed by Venkatesh et al. (4) elaborates on the determinants of technology adoption through the UTAUT model, emphasizing elements like perceived usefulness, effort expectancy, and facilitating conditions. This framework has been widely applied to understand user engagement with digital health tools and is particularly relevant for discussions around mHealth applications.

The growing concern over college students’ mental health, with rising rates of stress, anxiety, and depression, underscores the urgency for innovative interventions (5). The negative impacts of poor mental health—including diminished academic performance, lower sleep quality, and increased dropout rates—highlight the need for practical tools to support student well-being (6, 7). mHealth applications have gained traction due to their convenience, personalization, and ability to empower users to take control of their health (8). However, despite their potential, few studies have specifically examined college students’ perspectives on such apps’ design, usability, and overall value (9, 10). This qualitative study explores college students’ perceptions of mHealth well-being applications, focusing on the proposed AI-driven application. Through focus groups, we sought to gather insights into students’ attitudes, expectations, and preferences regarding the design and usability of an AI-based system aimed at promoting well-being.

## Methodology

This study employed a qualitative research design using focus groups to explore college students’ perceptions of the proposed mHealth application. The focus groups were conducted by two Master of Public Health (MPH) students at a public university in southeastern Virginia and facilitated by the Department of Health Behavior, Policy, and Management. This study was determined to be exempt from IRB review under Exemption Category #2 by the Institutional Review Board. The research was conducted in accordance with federal regulations for the protection of human participants. A total of 20 undergraduate students participated in five focus groups, one of which was conducted online.

**Figure 1:**
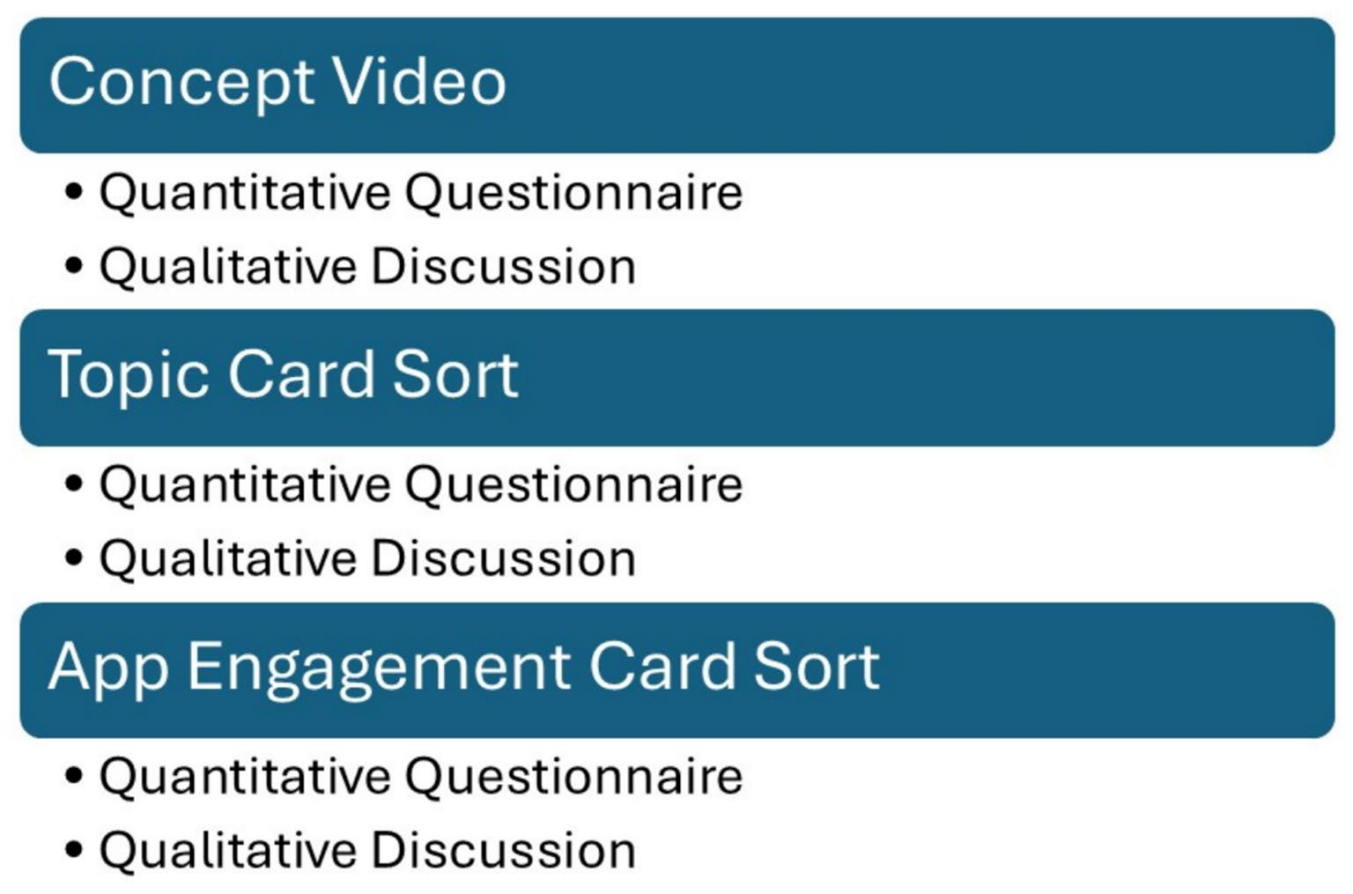
Focus group tasks and discussion.

Participants were recruited through convenience—departmental and course emails, face-to-face invitations at the student center, and professor referrals. Informed consent was secured prior to agreeing to participate in the study by all students. A facilitation guide and training were provided to the two MPH students who led the focus groups. Each focus group lasted approximately two hours, included four to five students, and followed a structured sequence of activities (Figure 1) designed to elicit feedback on proposed features and user experience. The session began with a concept video introducing the mHealth application, followed by a pre-process questionnaire that captured participants’ initial reactions. Students then engaged in card sorts, ranking various topics and interaction preferences relevant to the proposed app. Discussions following each card sort activity allowed participants to explain their rankings and provide deeper insight into their preferences for app features, interaction frequency, and overall usability. After the cards were sorted, a post-process questionnaire collected further feedback on the concept. Two weeks after the five focus groups concluded, participants were sent a follow-up questionnaire to assess the recall of critical topics and their likelihood of using and recommending the proposed app to others. There was no loss to follow-up.

Data triangulation was achieved using multiple data sources throughout the five focus groups, from the concept video to the topic and engagement card sorts, pre- and post-process questionnaires, and the recall survey. Group discussion after each activity allowed for alignment and saturation in an iterative and structured process. Layering diverse data collection methods is a technique that uses repetition and comparison to elicit both expected and novel insights. In addition, concurrent, content analysis using NVIVO v.12 was performed after each focus group session by reviewing transcripts from audio recordings and notes to identify when categories were well-developed and no new information was being shared. The lead author and the MPH students performed the data analysis.

## Results

The focus group discussions provided valuable insights into college students’ attitudes toward an AI-assistant for well-being. The primary theme was an overarching interest in managing personal health and wellness amid work, school, and relationship pressures. Students expressed a strong desire for tools to help them achieve and sustain a balanced life, particularly as they transition into their professional careers.

### Life balance and financial stability

Achieving work-life balance was a recurrent theme. Most students emphasized the challenges of maintaining this balance while juggling multiple responsibilities. Financial wellness emerged as a crucial aspect of well-being, closely linked to stress management and future stability.

“Right now, I feel like I kind of put my own wellness on the backburner a lot of the time to prioritize other things like school, especially being in my penultimate semester. So, it is very important, because it allows me to achieve my goals. And without my own well-being I would not be able to get to where I want to be.”—Focus Group #3.

“I will say financial stability. I think that’s my number one because after everything has been going up.. gas and like groceries and stuff, like people are trying to figure out what they are gonna do. Financial stability like that should be—not everyone’s number one, but top three.”—Focus Group #1.

### Privacy and community support

Students highly valued community and social support but preferred these interactions to be collaborative rather than competitive. Most participants appreciated the privacy offered by digital platforms, noting that face-to-face discussions around sensitive topics could be intimidating or judgmental.

“Especially in today’s digital age, after COVID too, especially people are so much more accustomed to being behind the screen, and not having to be face to face with those people.. We are so much more used to that. And we do not need that physical interaction to satisfy that need.”—Focus Group #3.

“There were a few cards that mentioned like community competition and like reminders and stuff like that and I’m just not a very like competitive person so I do not think I would use this app to be like oh, this now it’s like a competition of who can be better.. it would be more so like I know I just want to better myself and be more involved in the community, but not in a competitive way.”—Focus Group #2.

### Desired AI-assistant features

Participants expressed a strong interest in a user-friendly app that prioritizes personalization. Features of interest included customizable reminders, daily check-ins, and progress tracking. They also valued educational content, social media integration, and a comprehensive platform integrating physical, mental, and financial well-being.

“The daily check-ins because that’s something that can be really simple and quick and just it can provide information to go into more in-depth conversations later on.”—Focus Group #2.

“I like to track everything, and it keeps you honest, like you are actually paying attention.. Being able to see your progress like being able to look back and be like wow, I’ve really improved since then.”—Focus Group #4.

Therefore, the proposed application would provide students with evidence-based recommendations that lead them to adopt behaviors that scientific evidence shows will improve their health and academic performance. The application would offer specific recommendations for four essential lifestyle areas, which include physical activity, dietary habits, social interactions, and personal development. The proposed application would unite behavioral science principles with user-centered design approaches. The application will help students adopt sustainable behaviors that promote their educational success and overall well-being.

The primary limitation of this study is a small sample size of 20 undergraduate students from various degree programs across the University. The focus groups confirmed the proof of concept for the AI-assistant’s utility and feasibility influencing prototype development.

## Discussion

The results highlight the critical need for a personalized, user-friendly AI assistant designed to help college students manage their holistic well-being. Students seek tools that integrate physical, mental, and financial health into a single, easy-to-use platform. They also value features like customizable reminders, educational content, and engaging in a supportive, non-competitive community.

A key challenge for the app will be to balance personalization with simplicity. The app must provide meaningful support without overwhelming users with frequent notifications or complicated features. Additionally, while social media and technology integration are essential for reaching the target audience, these features must be implemented thoughtfully to avoid adding unnecessary stress.

Research has identified multiple obstacles which affect the development of mHealth applications. The development of functional and secure mHealth applications faces obstacles from inadequate security standards and restricted stakeholder participation according to Aijedaani and Barbar (11). The 2023 review of mHealth applications emphasizes the need for iterative user-centered design to achieve a balance between advanced functionalities and overall usability (12). The research builds upon previous studies by showing that college students need strong security measures and privacy protection, together with advanced features that do not overwhelm them and improved usability. Students expressed interest in work-life balance and financial stability because these two themes appeared as stressors and future stability indicators.

As the field continues to evolve rapidly, ongoing comparison with emerging studies will be essential for sustained growth. The proposed application appears to align well with student preferences. However, future research will be necessary to confirm its perceived usefulness, required effort, and facilitating conditions as outlined by the UTAUT framework. Additional studies could also address unresolved challenges, such as the deeper integration of real-time feedback loops and the use of advanced data analytics to further personalize interventions.

## Conclusion

College students expressed a strong interest in using an AI-driven app to help manage their health and well-being. These findings suggest focusing on user-centric design, personalization, and comprehensive support. This research suggests these ideas have the potential to become a valuable resource for students seeking balance, support, and long-term well-being. Future research should explore whether these design preferences translate into sustained user engagement and improved health outcomes.

## Data Availability

The raw data supporting the conclusions of this article will be made available by the authors, without undue reservation.
